# CAR T Cell Therapy: A Game Changer in Cancer Treatment

**DOI:** 10.1155/2016/5474602

**Published:** 2016-05-19

**Authors:** Hilde Almåsbak, Tanja Aarvak, Mohan C. Vemuri

**Affiliations:** ^1^Cellular Medicine, Bioproduction, Thermo Fisher Scientific, 0309 Oslo, Norway; ^2^Cell Biology, Thermo Fisher Scientific, Frederick, MD 21704, USA

## Abstract

The development of novel targeted therapies with acceptable safety profiles is critical to successful cancer outcomes with better survival rates. Immunotherapy offers promising opportunities with the potential to induce sustained remissions in patients with refractory disease. Recent dramatic clinical responses in trials with gene modified T cells expressing chimeric antigen receptors (CARs) in B-cell malignancies have generated great enthusiasm. This therapy might pave the way for a potential paradigm shift in the way we treat refractory or relapsed cancers. CARs are genetically engineered receptors that combine the specific binding domains from a tumor targeting antibody with T cell signaling domains to allow specifically targeted antibody redirected T cell activation. Despite current successes in hematological cancers, we are only in the beginning of exploring the powerful potential of CAR redirected T cells in the control and elimination of resistant, metastatic, or recurrent nonhematological cancers. This review discusses the application of the CAR T cell therapy, its challenges, and strategies for successful clinical and commercial translation.

## 1. Introduction

**Figure 1:**
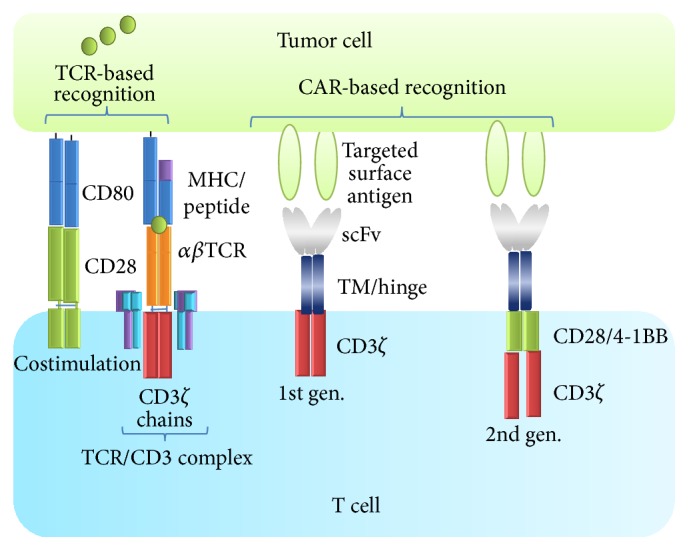
Elements involved in TCR and CAR recognition and activation. The TCR is disulfide-linked heterodimer consisting of one *α* and one *β* chain expressed in complex with invariant CD3 chains (*γ*, *δ*, *ζ*, and *ε*). The TCR recognizes intracellular or extracellular proteins presented as peptides by MHC molecules. Costimulation of CD28 through its ligands, CD80/CD86, is required for optimal activation and production of interleukin-2 (IL-2) and other cytokines. While most hematological tumors express costimulatory molecules, solid tumor cells as well as antigen presenting cells in the tumor microenvironment usually lack such molecules. CARs recognize surface antigens in an MHC unrestricted manner. CARs are fusion proteins between single-chain variable fragments (scFv) from a monoclonal antibody and one or more T cell receptor intracellular signaling domains. Various hinges and transmembrane (TM) domains are used to link the recognition and the signaling molecules [5]. While first generation CARs signaled through the CD3*ζ* chain only, second generation CARs include a signaling domain from a costimulatory molecule, for example, CD28 (illustrated), 4-1BB, OX40, CD27, or ICOS.

Chemotherapy and radiation have long been the mainstay of nonsurgical cancer treatment options. However, many cancers remain refractory to treatment and develop resistance to treatment modalities over time. Despite recent therapeutic advances, such as the introduction of monoclonal antibodies and small-molecular inhibitors, treatment responses vary considerably among patients and a high relapse rate with poor prognosis continues to be a major challenge. In case of persistent or relapsed disease, few or no treatment strategies are capable of definitely eradicating residual malignant cells, necessitating therapies with greater efficacy. Overwhelming evidence supports the critical role of the immune system, and lymphocytes in particular, in controlling and eradicating cancer. Harnessing the immune system to achieve clinical efficacy has been the focus of many therapies. More than two decades have passed since Gross and colleagues first demonstrated the principle of genetically redirecting cytotoxic T lymphocytes to tumor cells and concluded their seminal work with the statement that* chimeric T cell receptors with antitumor specificity will enable testing feasibility of this approach in combating human tumors *[1]. This study laid the foundation for the development of a series of first generation CARs where a tumor targeting antibody single chain variable fragment (scFv) is fused directly to the signaling domain of the T cell receptor (TCR) signaling complex member CD3*ζ* (Figure 1). Despite high target-cell specific killing* in vitro* and encouraging preclinical efficacies in murine tumor models, clinical responses of adoptively transferred T cells expressing *α*-folate receptor (FR) specific CAR in ovarian cancer were disappointing [2]. No reduction of tumor burden was seen in the 14 patients studied. The absence of efficacy was ascribed to lack of specific trafficking of the T cells to tumor and short persistence of the transferred T cells. First generation CARs deliver the primary activation signal to the T cells (signal 1) but the activated T cells are susceptible to anergy or activation induced cell death (AICD) in the absence of exogenous costimulation (signal 2) and fail to persist* in vivo* [3]. Further, T cells were expanded* ex vivo* for up to 56 days with partially insufficient costimulation, a lengthy process known currently to reduce the numbers of less-differentiated cells that maintain proliferative capacity and produce a continuous source of effector progeny after adoptive transfer [4].

Years of successive and significant innovations have finally culminated in clinical studies demonstrating the tremendous potential of second generation CAR expressing T cells (Figure 1). Genetic redirection of patient T cells with CARs targeting the B lymphocyte antigen CD19 has met with exceptional success in various therapy-refractory hematologic diseases (reviewed in [9]). Given their remarkable activity, CAR T cells are expected to enter the mainstream of health care for refractory or relapsed B-cell malignancies within few years and become the game changer for similar approaches in treating other cancers, such as solid tumors. Recent achievements result from novel molecular and immunological insights and provide the basis for further improvements of T cell therapies by driving consecutive developments of CAR design, optimization of T cell manufacturing, and incorporation of patient preconditioning and suggest novel treatment combinations [10].

## 2. T Cell Therapy in Cancer

The efficacy of adoptive T cell therapy (ATC) in human cancers was first demonstrated by the induction of molecular remission after donor lymphocyte infusion (DLI) in myeloid malignancies relapsing following bone marrow transplantation [11, 12]. Further studies demonstrated that expanded tumor infiltrating lymphocytes (TIL) could induce complete, long-lasting regression of large vascularized metastatic melanomas [13–15]. ATC using Epstein-Barr virus- (EBV-) specific T cells showed clinical benefit in various EBV-associated malignancies, including Hodgkin's disease, Burkitt's lymphoma, and nasopharyngeal carcinoma [16–18]. In addition, circulating tumor-reactive T cells from patient's peripheral blood, when* ex vivo* expanded in sufficient quantity and administrated to the patients, showed clinical benefit [19]. While these therapies rely on the endogenous T cell repertoires, recent technological advances in T cell engineering with retroviral and plasmid vectors allow the generation of high numbers of tumor targeting T cells by genetically introducing tumor specific T cell receptors (TCR) or CARs (Figure 1). In contrast to TCRs which recognize peptides derived from cellular proteins presented in the context of major histocompatibility complex (MHC), the more universally applicable CARs exhibit high-affinity MHC independent recognition of, in theory, any surface antigen, including carbohydrates and phospholipids [20–23].

**Figure 2:**
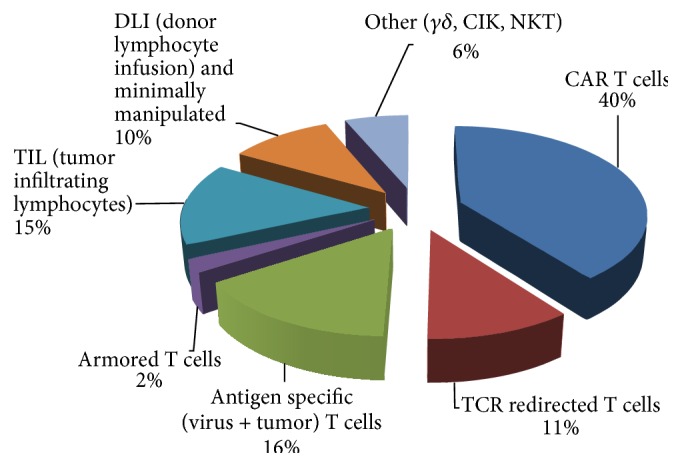
Open clinical studies investigating the safety and efficiency of adoptive T cell therapy (ATC) in cancer registered in https://clinicaltrials.gov/ as of December 2015 (search terms: “intervention: T cells”, indication: “cancer”). More than 200 protocols are registered, and about 40% of these address the use of CAR T cells, of which 65% are studied in trials for hematological malignancies. The use of unmodified/minimally manipulated/nongene modified T cells (based on the endogenous T cell repertoire) isolated from PMBC and from tumor (TILs) constitutes a similar fraction (16% virus/antigen specific + 15% TIL + 10% DLI/minimally manipulated) to CAR T cells. TCR gene modified T cells make-up about 11% of the studies. The term armored T cells refers to the adoptive transfer of T cells that have been precoated* ex vivo* with bispecific antibodies targeting CD3 and tumor associated antigen, like CD19 [6].

The number of open ATC studies in cancer registered in https://clinicaltrials.gov/ is rapidly increasing; as of December 2015 there are more than 200 protocols with the enrollment of more than 8000 patients worldwide [24]. About 40% of the protocols address the use of CAR T cells (Figure 2) with most trials (85%) being conducted in US and in China [25]. About 65% of the studies are directed against hematological malignancies [26, 27]. While CD19 is by far the most common antigen targeted in hematological B-cell cancers (>80%), studies are underway to investigate other target antigens such as CD20, CD22, CD30, ROR1, *κ* light chain, CD123, CD33, CD133, CD138, and B-cell maturation antigen [28–30]. Although solid tumors were the first targets of CAR T cell therapies [2, 31], realistic clinical responses are seen in clinical studies where patients with various B-cell malignancies have been treated with CD19 CAR T cells [9]. One of the first encouraging reports came from investigators at the National Cancer Institute (NCI) which published a case study in 2010 where a heavily pretreated patient with follicular lymphoma experienced a dramatic partial remission (PR) after receiving preconditioning chemotherapy followed by infusion of T cells retrovirally transduced to express a second generation CD19 CAR with a CD28 costimulation domain [32]. Shortly after this breakthrough, June's group at the University of Pennsylvania (UPENN) presented early clinical results showing impressive antileukemia efficacy of T cells transduced with a lentiviral vector carrying a CD19 CAR with a 4-1BB costimulation domain [33, 34]. Complete remissions (CR) were seen in two of the three treated patients with end-stage advanced chronic lymphocytic leukemias (CLL) and a partial response in the third patient. The results after complete enrollment of the trial were recently published and reported an overall response rate of 57% with 4 out of 14 treated patients in CR and 4 PRs [35]. Importantly, the study demonstrated that the sustained capability of the CAR T cells to expand* in vivo* correlated with clinical responses. Furthermore, CAR T cells persisted and remained functional beyond 4 years in the first two patients achieving CR, with no relapse. CD19 CAR T cell function and engraftment might be improved further when combined with ibrutinib, which is a small-molecular inhibitor of the enzyme Bruton's tyrosine kinase (BTK) associated with increased B-cell activation and proliferation [36]. The far most remarkable responses with CD19 redirected T cells have been reported by groups at UPENN, Memorial Sloan Kettering Cancer Center (MSKCC), and NCI in patients with resistant or relapsed acute lymphoblastic leukemia (ALL) with CR rates ranging from 70 to 90% in approximately 65 patients among the three trials, combined [37–39]. More recently, investigators at Great Ormond Street Hospital and University College London Institute of Child Health's treated a 1-year-old girl with ALL who had relapsed shortly after bone marrow transplantation (BMT) with* off-the-shelf* banked CD19 CAR redirected allogeneic T cells derived from a healthy donor (UCART19) [40]. While the treatment resulted in cytogenetic and molecular remission of her leukemia, a second BMT given 3 months after the T cell injection precludes the interpretation of the long-term efficacy of the UCART19 therapy. The infused allogeneic CAR expressing T cells were gene edited by nucleases to disrupt expression of the endogenous TCRs to avoid alloreactivity [41]. Studies have clearly demonstrated that allogeneic CAR T cells can not only induce tumor regression but also drive GVHD [42, 43] and the UCART19 strategy is therefore critically dependent on high TCR knockdown efficacy or efficient depletion of TCR expressing T cells prior to infusion. Of interest is also a case report of a multiple myeloma patient in CR after CD19 CAR T cell therapy despite lack of detectable CD19 expression in 99.95% of the patient's neoplastic plasma cells [44]. The response is hypothesized to be caused either by elimination of a small population of CD19 expressing myeloma stem cells or by elimination of CD19 expressing cells that play a critical role in sustaining the growth of the myeloma cells. Encouraging clinical results have also been obtained in patients with various chemotherapy refractory B-cell lymphomas, including CR in four out of seven evaluable patients with diffuse large B-cell lymphoma (DLBCL) after infusion of CD19 CAR T cells [45]. By contrast, it has been difficult to see the clinical efficacy of CAR T cells in nonhematological, solid tumors. By targeting the disialoganglioside GD2 expressed on neuroblastoma with CAR T cells, investigators at Baylor College of Medicine report some clinical benefit with CR in three of eleven patients with active disease [46]. Among open clinical protocols for solid tumors, CARs targeting mesothelin, which is overexpressed in a wide range of solid tumors [47], human epidermal growth factor receptor family members (HER2/ERBB2 and HER1/EGFR) overexpressed in breast, ovarian, bladder, salivary gland, endometrial, pancreatic, and non-small-cell lung cancer (NSCLC) [48–51], and neuroblastoma associated GD2 [52] antigens dominate. However, increasing number of targets is being investigated in clinical trials, such as MUC1 and carcinoembryonic antigen (CEA) overexpressed in various carcinomas, fibroblast activation protein (FAP) targeting cancer associated fibroblasts in the tumor stroma, and vascular endothelial growth factor receptor 2 (VEGFR2) overexpressed in tumor vasculature [23, 53–57].

## 3. Factors Affecting Efficacy of CAR T Cell Therapy

**Figure 3:**
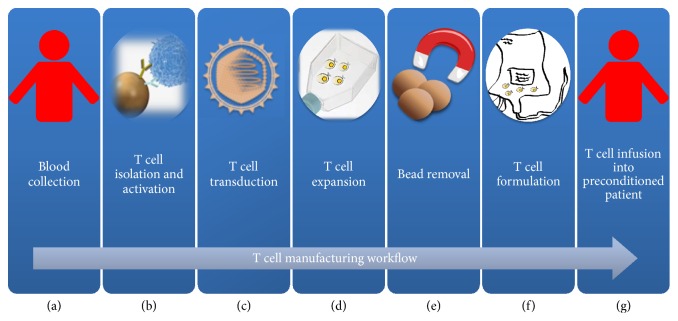
Example of manufacturing and delivery pipeline of CAR T cell therapies [7, 8]. Peripheral blood mononuclear cells (PBMCs) are harvested from the patient (or a T cell donor) (a) and transferred to a good manufacturing practice (GMP) facility, where the T cells are isolated and activated in the presence of magnetic beads conjugated with CD3 and CD28 antibodies (b) and subsequently genetically engineered by viral transduction to express the CAR (c). The activated T cells are expanded* ex vivo* for a period, typically 10–14 days, to reach a therapeutic relevant number (d) before magnetic bead removal (e) and formulation, either for freezing or for adoptive transfer (f). The patient undergoes a conditional chemotherapy prior to infusion of the CAR T cells (g).

Many known and numerous yet unidentified factors are likely to contribute to the variability observed in clinical responses across trials and also between individual patients. Despite the fact that differences in clinical protocols preclude direct comparisons, clinical data collectively point at T cell expansion and persistence after adoptive transfer as key critical factors for achieving an effective clearance of the cancer [9]. The* in vivo* fate of the T cells is influenced by several factors broadly related to the CAR design, the composition of the infused T cells, the tumor type and microenvironment, and recipient preconditioning regimen. Savoldo and colleagues elegantly demonstrated the significance of introducing costimulatory domains into second generation CARs by treating six lymphoma patients with a mixture of first and second generation CAR T cells, providing evidence for the enhanced persistence of T cells expressing the latter CAR configuration [58]. Most second generation CARs studied in clinical trials incorporate CD28 or 4-1BB signaling domains and preclinical and emerging clinical experience suggest that CD28 containing constructs undergo a more rapid expansion and subsequently decline, whereas 4-1BB CARs confer longer persistence [10, 59]. Third generation CARs incorporating CD28-4-1BB or CD28-OX40 in combination have demonstrated sustained activation of T cells [60–64] but their effectiveness remains to be evaluated in clinical trials. A clinical study utilizing a CD20-redirected third generation CD28-4-1BB-CD3*ζ* signaling CAR did not show dramatic responses [65]. Zhao and colleagues recently demonstrated superior efficiency of combining CD28 and 4-1BB signaling in a novel receptor configuration that provides CD28 costimulation through the endodomain in the CAR and 4-1BB costimulation by expression of the 4-1BB ligand, which is coexpressed at the cell surface with the CAR [5]. The* in vivo* proliferative capacity depends further on the composition of the T cells in the infused product. Long-term persistence and function are provided by central memory phenotype T cells that retain longer telomeres and higher proliferation compared to the more differentiated effector T cell populations [4, 66, 67]. Most clinical trials performed today utilize unselected,* ex vivo* expanded T cells obtained from patient peripheral blood mononuclear cells (PBMC). The use of paramagnetic beads covalently conjugated with agonistic CD3 and CD28 antibodies, such as CTS*™* Dynabeads*™* CD3/CD28, in combination with the CTS*™* DynaMag magnet adapted for culture bags, has been successfully implemented in the clinic as they allow for simultaneous isolation and activation of T cells from the PBMC [7, 68] (Figure 3). Short duration, in general around 10 days, of the* ex vivo* expansion results in a final T cell drug consisting of both CD4 and CD8 T cells displaying early memory phenotypes with the ability to expand in the blood of patients and generate long-term memory [33]. More recently, methods to isolate defined T cell subset under good manufacturing (GMP) conditions have been developed with the aim to better control the phenotype of the transferred T cells [69]. In a murine tumor model of lymphoma they demonstrated superior efficiency using a CAR T cell formulation consisting of CD4 T cells derived from the naïve CD4 T cell pool with CD8 T cells derived from central memory CD8 T cells at a 1 : 1 ratio, compared to unselected batch T cells and CD8 or CD4 cells alone [70]. Memory stem T cells [71, 72], ICOS costimulated Th17-polarized T cells [73–76], and virus specific memory T cells [77–79] have also attracted interest as effective T cell populations with great replicative potential. One factor that has been shown to impact T cell engraftment and proliferation is the use of lymphodepletion chemotherapy in patients prior to T cell infusion [14, 80]. This preconditioning creates space for the expansion of infused cells, limits the competition for homeostatic gamma chain cytokines IL-7 and IL-15, depletes regulatory T cells, and activates the innate immune system. Finally, relapse with CD19 negative tumor cells after CAR T cell therapy remains a challenge [81]. Single-target therapy may select for and lead to escape of the variants and targeting multiple antigens on tumors would increase the chances of therapeutic efficiency. Combination of CARs with different specificities, or the use of bispecific tandem CARs, which join two antigen-recognition moieties, may prevent relapses due to escape of variants but require further studies [82].

While solid tumors have proven largely refractory to T cell therapy, encouraging preclinical and clinical data support further development. Solid tumors are challenging; their microenvironment is extremely inhospitable and induces T cell anergy [83]. Technological advances required to enhance CAR T cell function and survival in solid tumors include strategies to increase T cell trafficking, T cell resistance to the immunosuppressive environment, and recruitment of other immune effectors (reviewed in [84–86]). T cells must survive and overcome an environment characterized by oxidative stress and hypoxia, the presence of suppressive immune cells and factors, and T cell intrinsic negative regulatory mechanisms including upregulation of inhibitory receptors. CAR design has been configured to create “Trucks” (T cells redirected for universal cytokine-mediated killing) and “Armored CARs” that express cytokines and chemokine receptors and recently also modified to coexpress catalase to protect the T cells from oxidative stress-mediated repression and heparanase to improve T cell penetration through tumor stroma and enhance infiltration [87–90]. Combination of ATC with checkpoint inhibitor blockade using antagonistic antibodies against the negative regulators CTLA-4 and PD-1/PD1-L has also been suggested, and it has been demonstrated that the specific blockade of the PD-1 immunosuppressive pathway significantly enhanced the function of HER2 redirected CAR expressing T cells leading to enhanced tumor eradication in immune competent HER2 transgenic mice [91]. Recently, studies have also demonstrated promising potential of T cells gene modified with fusion receptors comprising the extracellular domain of PD-1 linked to the cytoplasmic domain of CD28 in reversing inhibitory effects of PD-1 binding [92, 93].

## 4. Factors Affecting Safety of CAR T Cell Therapy

Given the extreme potency of CAR modified T cells, the use of this therapy has significant toxic potential [94–96]. Toxicities range from life threatening cytokine release syndromes (CRS) and macrophage activation syndromes (MAS) to on-target off-tumor toxicity, neurotoxicity, and tumor lysis syndrome (TLS). CRS and neurotoxicity appear to be frequent in B-cell malignancies but are in most cases treatable and reversible [38, 97]. CRS is associated with high circulating levels of several cytokines, including interleukin-6 (IL-6) and interferon-*γ*, and seems to correlate with high antitumor activity and high tumor burden. CRS is frequently accompanied by MAS, which may partly be driven by elevated levels of IL-6 [98]. Both CRS and MAS can be mitigated by infusion of the monoclonal antibody tocilizumab which blocks the action of IL-6 and reduces inflammation [37]. The mechanisms underlying the neurologic symptoms including aphasia, tremor and seizures remain poorly understood; however, it has been reported that MAS can be associated with neurological toxicity [9]. On-target off-tumor toxicity of CAR modified T cells was first reported in a phase I clinical trial of renal cell carcinoma patients treated with T cells expressing a CAR recognizing carbonic anhydrase IX (CAIX) [31]. Here, several patients experienced significant liver toxicity due to the expression of CAIX on normal bile duct epithelium, necessitating cessation of treatment. The first fatal adverse event due to off-tumor recognition by a CAR occurred in a patient with colorectal cancer treated with high numbers of T cells expressing a third generation CAR targeting ERBB2/HER2 [95]. The patient developed respiratory distress and cardiac arrests shortly after the T cell transfer and died of multisystem organ failure 5 days later. It was postulated that the CAR T cells recognized ERBB2 expressed at low levels in the lung epithelium, leading to pulmonary toxicity and a cascading cytokine storm with a fatal outcome. Predicted on-target off-tumor toxicity with depletion of normal B-cells has been reported in nearly all patients treated with CD19 CAR T cells, and depending on the CAR configuration, B-cell aplasia lasts from months to years [35, 39]. To mitigate this toxicity, patients receive monthly immunoglobulin replacement; however, long-term follow-up is needed to assess the late effects of B-cell aplasia. Because few CARs are truly tumor specific but recognize both normal and malignant cells, strategies to improve specificity are warranted. Affinity-tuned CARs based on low-affinity scFv recognition have been demonstrated to increase tumor specificity for targets that are overexpressed compared to normal tissues expressing the same target at physiological levels [99]. Furthermore, various dual targeting strategies have been developed to increase specificity and safety. One strategy is based on T cells modified with two different CARs, where CAR number one provides the CD3*ζ* signal and initiates killing, whereas CAR number two transmits the costimulation signal [100–102]. Full CAR T cell activation and function are only achieved when the T cell is engaged by both CAR antigens. Moreover, inhibitory CARs (iCARs) that harness natural T cell inhibition exerted by PD-1 and CTLA-4 have been demonstrated to protect normal tissue from off-target effects in preclinical mouse models [103]. The inhibitory function of the iCAR T cell is a result of checkpoint inhibition initiated in response to an antigen found on normal tissue but not on the tumor. Other approaches are based on switchable CARs (sCAR) and multichain CARs (mcCARs) that are activated only in the presence of intermediate switch molecules [104, 105]. While the sCAR design is based on coinfusion of antibody-based switch molecules bridging the target cell and the sCAR expressing T cell, mcCARs are fully activated only in the presence of the small-molecule drug, such as rapamycin. The switch approach has been used to achieve reversible control of sCAR T cell activity in immunocompetent mouse model of CD19 targeting [106]. The sCARs principle further allows for simultaneous targeting of several tumor antigens simply by infusion of switch molecules conferring two or more specificities, for example, CD19 and CD22. The severity of chronic toxicities can be mitigated by introducing suicide genes in the vector used for CAR gene transfer [107–109] or allow surface coexpression of binding epitopes for depleting antibodies already in clinical use, for example, EGFR and CD20 [110, 111]. Other approaches rely on the use of self-limiting, transiently expressed CARs [56, 112, 113] or administration of blocking antibodies and steroids [114]. Finally, integrating vectors used to facilitate the CAR gene transfer into T cells might constitute a safety risk in the clinical setting as it raises the theoretical possibility of insertional mutagenesis as demonstrated in stem cell gene therapy studies in primary immunodeficiencies [115]. Despite the fact that numerous studies with more than 500 patient-year follow-up have demonstrated the safety of retroviral gene transfer into mature T cells [116, 117], it is too early to conclude that integration is safe in a larger patient population and effective strategies are needed to eliminate gene modified T cells.

## 5. Conclusions and Future Perspectives

The adoptive transfer of gene modified T cells is a rapidly evolving innovative treatment for cancer. CAR redirected T cells are renewable drugs with the capacity to proliferate in the patient after infusion and further to persist and provide sustained functional immunity. The efficacy has been demonstrated in a range of hematological cancers including ALL, CLL, DLBCL, FL, and multiple myeloma [26] and further by some encouraging clinical data reported in early phase I trials in solid tumors, including neuroblastoma, and tumors overexpressing mesothelin, HER2, and EGFR [46, 50, 51, 56]. The clinical successes with CD19 CAR T cells in leukemia and lymphomas have boosted the field and led to significant pharmaceutical and venture capital funding of the biotech sector, as well as promoting innovative academic-industrial partnerships to explore new discoveries in basic research that may translate into clinical and commercial development [118].

This rapidly developing field meets with considerable challenges which have to be addressed to realize the promise of the CAR T cell therapy for a broader use. While CAR T cell therapies have provided encouraging preliminary signs of efficacy in solid tumors, clinical data so far fail by a large margin to meet expectations for game-changing cell therapy. A major focus of translational research is to improve specificity, efficacy, and safety of CAR T cells to be used in cancers beyond leukemia. Truly tumor specific surface antigens are hardly identified, and the implementation of effective mechanisms to mitigate life threatening and unexpected off-target toxicities is crucial. Further, issues regarding tumor heterogeneity, tumor immunosuppression, and lack of T cell trafficking and persistence are being addressed to improve efficacy of solid tumor therapy [119]. Combining T cell therapies with immunomodulatory agents, for example, checkpoint inhibitors and cytokines, and/or small-molecular antagonists that block biochemical pathways crucial for tumor growth, constitute exciting opportunities that may have synergistic effects in augmenting antitumor responses.

Moreover, the CAR T cell technology must be commercialized at an acceptable cost. Pilot-scale processes for CAR T cell generation were originally developed in academic centers for early phase I clinical research, requiring small numbers of T cell products. These processes are based on manual and open-handling steps in safety cabinets and are not suited for commercial manufacturing of thousands of therapeutic T cell doses needed for a future approved therapy. Industrialized T cell processing can only be achieved with significant investments in automation and the establishment of a fully closed process [120]. Current industrial commercialization strategies are based on centralized T cell manufacturing facilities and a coordinated infrastructure to provide cost-effective cell-drug distribution [121]. An alternative strategy is based on a decentralized model where T cell manufacturing is performed within the treatment centers. Whether a centralized CAR T cell manufacturing will be more cost-efficient than utilizing the existing infrastructure (equipment, facilities, and competencies) in blood banks and clinics depends largely on successful process automation as well as on economics of scale [122] and on the other hand on the regulatory approach of the developing companies.

Improvements in gene modification [123], T cell selection, and expansion techniques, as well as the development of safe and more effective viral and nonviral vectors, will further enhance the integration of T cell gene therapies. Finally, to overcome the constraints associated with complicated logistics and manufacturing of the individualized T cell therapy in the autologous setting, significant efforts are under way to develop universal and off-the-shelf, allogeneic T cell drugs. While off-the-shelf T cells might allow more efficient manufacturing and reduce lead time for the administration of the T cell drug, there are some concerns regarding their use, including their potential to drive GVHD and their limited life span after transfer.

## Additional Points

*Glossary of Terms*. Donor lymphocyte infusion is the infusion of lymphocytes from the stem cell donor after stem cell transplantation to augment an antitumor (graft versus leukemia) immune response. Aplasia is the absence of cell type/organ lack of normal progression of cell generation.
